# Influence of TiO_2_@SiO_2_ hybrid nanoparticles on characteristics, fluidization behavior and coating performance of PA12/PPO blend

**DOI:** 10.1039/d6ra07570c

**Published:** 2026-09-29

**Authors:** Pham The Long, Nguyen Huu Dat, Tran Huu Trung, Mai Duc Huynh, Nguyen Thi Thu Trang, Nguyen Tien Minh, Hoang Thi Van An, Nguyen Thi Ngoc Quynh, Luong Nhu Hai, Nguyen Vu Giang

**Affiliations:** a High Technology Innovation Center, Vietnam Academy of Science and Technology 18 Hoang Quoc Viet, Nghia Do Hanoi 10000 Vietnam; b Institute of Materials Science, Vietnam Academy of Science and Technology 18 Hoang Quoc Viet, Nghia Do Hanoi 10000 Vietnam giangnv@ims.vast.ac.vn; c Graduate University of Science and Technology, Vietnam Academy of Science and Technology 18 Hoang Quoc Viet Street, Nghia Do Hanoi 10000 Vietnam; d Viet Tri University of Industry 9 Tien Son, Thanh Mieu Phu Tho 10000 Vietnam

## Abstract

In this study, TiO_2_@SiO_2_ (TS) hybrid nanoparticles were synthesized *via* the Stöber sol–gel method and incorporated into a PA12/PPO (90/10 wt%) blend at loadings of 1, 3, and 5 wt% by melt compounding, for application as a fluidized bed coating (FBC) material. The core–shell architecture of the TS nanoparticles was confirmed by field-emission scanning electron microscopy (FE-SEM), Fourier-transform infrared spectroscopy (FT-IR), and energy-dispersive X-ray (EDX) analysis. Among all formulations tested, the 3 wt% TS nanocomposite (TS-3) exhibited the most favorable balance of coating properties, surpassing the neat PA12/PPO blend and the equivalent TiO_2_ nanocomposite (T-3) in tensile strength, abrasion resistance, and coating flexibility. FE-SEM revealed a fibrous fracture texture in TS-3, compared with interfacial voids around neat TiO_2_ particles, suggesting differences in polymer–filler adhesion. Hydrogen bonding between silanol groups of the SiO_2_ shell and amide groups of PA12 is proposed as a possible contribution to these interfacial interactions. A notable finding of this study is that neat TiO_2_ lowered the peak decomposition temperature relative to the unfilled blend, possibly through surface-mediated promotion of thermal chain scission involving Lewis-acidic sites; the SiO_2_ shell may limit such interactions, while the TS-3 nanocomposite exhibited a temperature at 10% mass loss 25.7 °C higher than that of the neat blend. Dynamic mechanical analysis confirmed substantial positive shifts in the glass transition temperatures of both the PA12 and PPO phases for the TS-3 nanocomposite, reflecting strong polymer–filler interfacial interactions. The TS-3 FBC also exhibited lower thickness variability and higher surface gloss than the unfilled blend and its TiO_2_-filled counterpart. These results establish the PA12/PPO/TS nanocomposite system as a multifunctional FBC coating material with simultaneously enhanced mechanical, thermal, UV-barrier, and surface appearance performance.

## Abstract 2

TiO_2_@SiO_2_ (TS) hybrid nanoparticles were synthesized *via* the Stöber sol–gel method and incorporated into a PA12/PPO (90/10 wt%) blend by melt compounding, for application as a fluidized bed coating.

## Graphical abstract

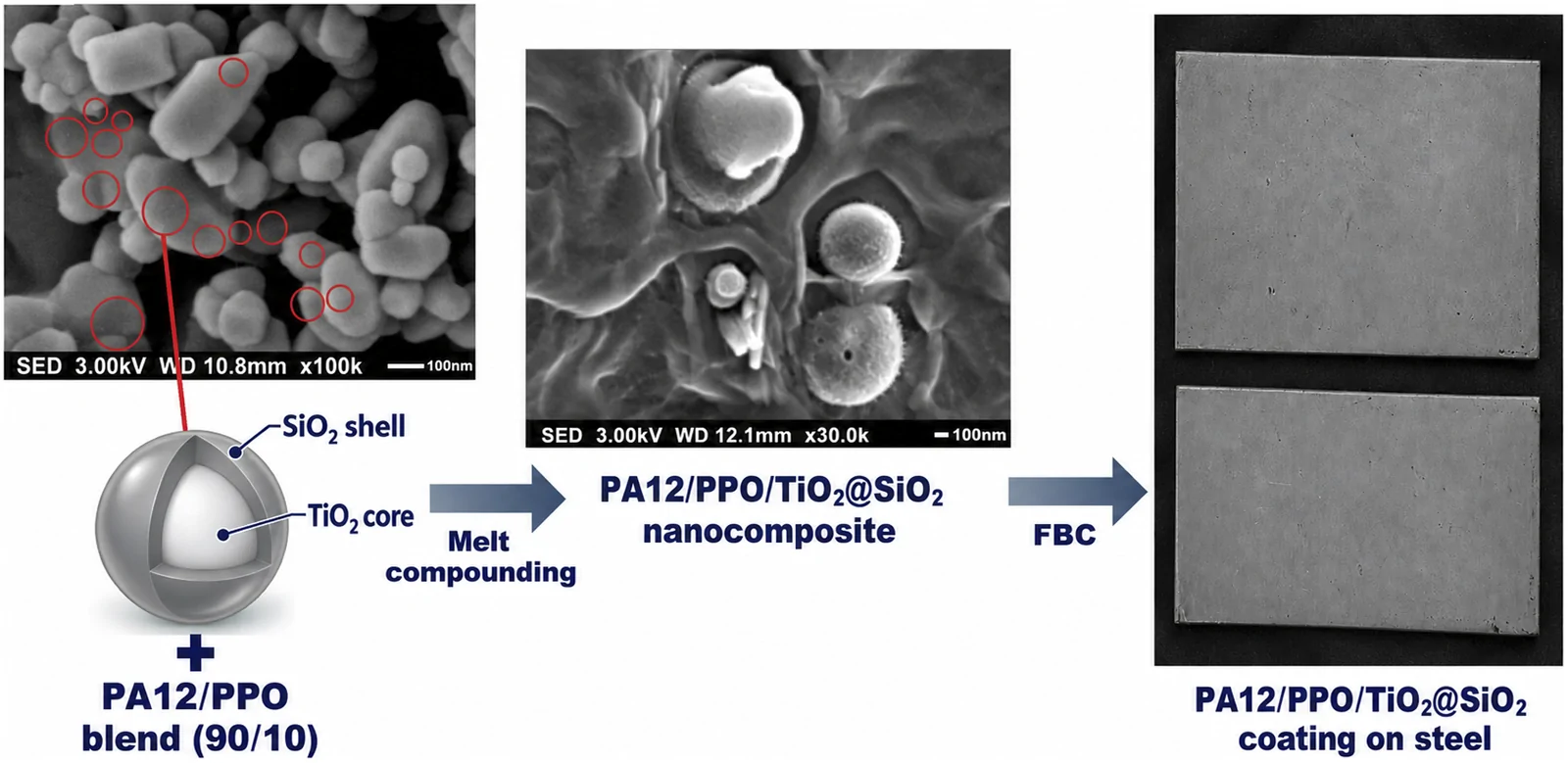

## 1 Introduction

Fluidized bed coating (FBC) is one of the most industrially established methods for applying thick thermoplastic coatings (200–500 µm) onto preheated metallic substrates.^1,2^ In the conventional hot-dipping FBC process, metal workpieces are preheated above the polymer melting point and then immersed into a fluidized powder bath, in which compressed air flowing through a porous distributor plate suspends the polymer particles in a pseudo-liquid state. Upon contact with the hot metal surface, the particles sinter and coalesce into a continuous, adherent coating film.^1^ Compared with solvent-borne liquid coatings, FBC delivers near-zero volatile organic compound (VOC) emissions, material utilization efficiencies exceeding 95%, and conformal coating of complex geometries without post-cure drying.^1,2^ These attributes have established FBC as the preferred protective coating technology in the automotive, pipeline, marine, electrical engineering, and appliance industries.^1,2^

Among thermoplastic powders used in FBC, polyamide 12 (PA12) has established itself as a widely used thermoplastic powder for high-performance applications.^3,4^ PA12 possesses the longest aliphatic chain among commercial polyamides (eleven –CH_2_– groups between successive amide linkages), conferring low moisture absorption (<0.5 wt% at 23 °C/50% RH), good chemical resistance to hydrocarbons, fuels, and saline solutions, high impact toughness at subzero temperatures, and good dimensional stability.^3,4^ Its relatively low melting temperature (∼178 °C) facilitates energy-efficient FBC processing, making it widely specified for coating metal pipe fittings, automotive fluid lines, and industrial components.^3^ Nevertheless, neat PA12 coatings suffer from limited heat distortion resistance, insufficient flame retardancy, and susceptibility to UV-induced photo-oxidative chain scission during outdoor service, restricting their applicability in high-temperature and UV-exposed environments.^3–5^

Polymer blending offers a practical and commercially viable route to simultaneously overcome multiple limitations of a single-component matrix without fundamentally altering the processing route.^6^ Poly(2,6-dimethyl-1,4-phenylene oxide) (PPO) is an amorphous engineering thermoplastic with a high glass transition temperature, inherent flame resistance (UL-94 V0 without halogenated additives), and high thermal stability.^7,8^ Blending PPO into a PA12 matrix has been shown to elevate the thermal stability and flame retardancy of the resulting material, as the high-*T*_g_ PPO domains restrict the segmental mobility of the PA12 chains and contribute a thermally stable aromatic char upon combustion.^8^ In our previous study, a PA12/PPO blend at a weight ratio of 90/10 was identified as the optimal composition, achieving a decomposition onset temperature and a UL-94 flammability rating upgrade from properties of neat PA12—while dynamic mechanical analysis confirmed partial miscibility through measurable glass transition temperature shifts in both component phases.^9,10^ These results establish the PA12/PPO (90/10) blend as a thermally and mechanically superior feedstock for FBC, offering improved heat resistance while retaining the processability and film-forming characteristics of PA12-rich systems.

A critical prerequisite for successful FBC is that the powder feedstock exhibits adequate flowability and a particle size distribution (typically 80–200 µm for PA-based systems) that ensures stable, homogeneous fluidization.^1,11^ According to the Geldart classification,^11^ FBC polymer powders ideally fall within Group B, characterized by bubbling fluidization above the minimum fluidization velocity with minimal channeling. Adding nanoscale particles to polymer FBC powders fulfills two simultaneous roles: (i) acting as flow conditioners that physically space host powder particles apart, reducing interparticle van der Waals cohesive forces and improving flowability and fluidization quality;^12,13^ and (ii) functioning as functional additives that improve the mechanical, thermal, optical, and surface properties of the deposited coating film.^14–16^ Yang *et al.* demonstrated that dry surface coating of cohesive polymer particles with Al_2_O_3_, SiO_2_, TiO_2_, and carbon black nanoparticles consistently reduced the angle of repose and minimum fluidization velocity of the host particles,^12^ making them potential additives for multifunctional FBC powder formulations.

Titanium dioxide (TiO_2_) nanoparticles are the dominant UV-absorbing white pigment in protective polymer coatings, owing to their very high refractive index (*n* ≈ 2.5 for rutile), strong UV absorption in the 300–400 nm range, and high visible-light reflectance.^17^ Their incorporation into polymer coating matrices increases pencil hardness, reduces water permeability, and improves corrosion protection.^14,16^ However, neat TiO_2_ nanoparticles suffer from two critical drawbacks: (i) pronounced inter-particle agglomeration driven by high surface energy, compromising both powder flowability in the FBC bed and nanoparticle dispersion within the deposited film; and (ii) strong photocatalytic activity under UV irradiation, which generates reactive oxygen species (ROS) that accelerate polymer chain scission and discoloration, counterproductively degrading the coating.^18^

Encapsulating the TiO_2_ core within an amorphous SiO_2_ shell—designated TiO_2_@SiO_2_ (TS) hybrid nanoparticles—addresses both limitations simultaneously. The dense, photocatalytically inert SiO_2_ shell physically blocks the active surface sites of TiO_2_, suppressing ROS generation while retaining the UV-absorbing capacity of the core; Bai *et al.* demonstrated that TiO_2_@SiO_2_/APTES nanoparticles retained ∼80% of the UV-shielding efficiency of uncoated TiO_2_ while reducing photocatalytic methylene blue degradation from ∼50% to only ∼4% under UV irradiation.^18^ More recently, hybrid TiO_2_@SiO_2_/organic UV-absorber systems exhibited zero photocatalytic activity and enhanced UV-shielding performance in polyurethane coating films sustained after more than 500 h of accelerated UV aging.^19^

In the context of FBC powder processing, the surface silanol (–Si–OH) groups of the SiO_2_ shell can interact with the host PA12/PPO powder particles during dry-blending, providing active sites that reduce van der Waals cohesive forces and improve the flowability of the composite powder feedstock for FBC.^12^ It must be noted, however, that these same silanol groups are themselves highly polar and prone to forming strong inter-particle hydrogen bonds between adjacent TS nanoparticles, which can drive self-agglomeration of the TS nanoparticles at elevated filler loadings in the absence of organic surface passivation.^12,18^ This competing tendency represents a practical upper bound on effective TS content in the blend—a consideration that informs the selection of the 1–5 wt% loading range investigated in the present study.

When incorporated into the PA12/PPO blend by melt compounding, TS nanoparticles are subject to selective localization governed by the relative surface energies of the nanoparticle surface and the two polymer phases. Depending on these thermodynamic parameters, TS particles may preferentially migrate to the PA12-rich phase, the PPO-rich dispersed phase, or—most favorably—to the PA12/PPO phase boundary, where they act as solid-particle emulsifiers (Pickering-type stabilizers) that sterically impede coalescence of the PPO dispersed domains, effectively compatibilizing the blend and refining its phase morphology. A Pickering-type interfacial stabilization by silica nanoparticles at the interface of PA/PPO blends has been reported,^9,20^ where functionalized SiO_2_ nanoparticles induced a transition from coarse droplet-matrix to finer, more uniform PPO domain morphologies. Additionally, a critical rheological challenge must be acknowledged: the strongly asymmetric melt viscosities of the PA12 matrix (lower viscosity) and the PPO dispersed phase (significantly higher viscosity) generate complex shear-stress fields during intensive melt mixing. While the high shear stresses can aid in breaking down TS nanoparticle agglomerates, they may simultaneously damage the structural integrity of the TiO_2_@SiO_2_ core–shell architecture, potentially exposing the TiO_2_ surface and partially restoring photocatalytic activity. Optimizing melt-compounding conditions—particularly screw speed and melt temperature—to balance dispersion efficiency with preservation of the core–shell structure is therefore an important processing consideration for this system.^18^

Despite the significant bodies of literature on PA12-based FBC and on TiO_2_@SiO_2_ hybrid nanoparticles in polymer systems separately, no study has yet investigated the combined effect of TiO_2_@SiO_2_ hybrid nanoparticles on a PA12/PPO blend in the context of FBC coating technology. The present work reports the synthesis, characterization, and FBC application of TiO_2_@SiO_2_/PA12/PPO nanocomposites, examining how the core–shell architecture of the hybrid nanoparticles influences the mechanical properties, thermal stability, phase morphology, and FBC coating quality—including thickness uniformity and surface gloss—of the deposited films on mild steel substrates.

## 2 Experimental

### 2.1. Materials

Polyamide 12 (PA12) with a melt flow index of 7.4 g/10 min (235 °C; 2.16 kg) and density of 1.03 g cm^−3^ was supplied by UBE Corporation, Japan. Poly(2,6-dimethyl-1,4-phenylene oxide) (PPO) with a bulk density of 0.465 g cm^−3^ was purchased from SABIC, Saudi Arabia. Rutile TiO_2_ nanopowder (with diameter of about 40–100 nm) provided by Merck Corp, Germany. Tetraethyl orthosilicate (TEOS, ≥99%), absolute ethanol (≥99.8%), and concentrated ammonia solution (NH_3_ H_2_O, 25–28 wt%) were obtained from Sigma-Aldrich and used as received.

### 2.2. Synthesis of TiO_2_@SiO_2_ hybrid nanoparticles

TiO_2_@SiO_2_ (TS) hybrid nanoparticles were synthesized using the Stöber sol–gel method,^21^ adapted for core–shell oxide nanoparticle preparation by direct deposition of a SiO_2_ shell onto TiO_2_ templates following previously reported procedures.^18^ Briefly, 1.44 g of TiO_2_ was dispersed in ethanol (240 mL) and deionized water (54 mL) by ultrasonication at 120 W for 30 min, followed by magnetic stirring for 30 min. Concentrated ammonia solution (6 mL, 25 wt%) was added and the mixture stirred for 30 min. TEOS (2.4 mL) was added dropwise under continuous stirring and the reaction continued for 12 h at room temperature. The product was aged 24 h, washed alternately with ethanol and deionized water, and dried at 60 °C for 12 h.

### 2.3. Preparation of PA12/PPO nanocomposites

Nanocomposites were prepared by melt compounding in a HAAKE internal mixer at 235 °C and 50 rpm. PA12/PPO (90/10 wt%) was first blended for 5 min, then neat TiO_2_ or TS nanoparticles (1, 3, or 5 wt%) were incorporated for a further 3 min. Sheet specimens (1.5–2.0 mm) were prepared by hot pressing at 250 °C and 10 MPa for 5 min. The melt-compounded nanocomposite blends were subsequently reduced to FBC-grade powder by cryogenic impact grinding, in which the material was pre-embrittled by immersion in liquid nitrogen (−196 °C) prior to pulverization in an impact mill.^23,24^ FBC-coated coupons were made by preheating mild steel substrates (100 × 100 × 3 mm) to 280 °C and immersing them in the fluidized powder bed for 5 s, then cooling in air.

### 2.4. Characterization

Tensile properties were evaluated according to ASTM D638 using Type IV dumbbell-shaped specimens (five specimens per formulation) at a tensile speed of 10 mm min^−1^ on a Zwick Tensiler 2.5 (Germany) device. Coating flexibility was evaluated by mandrel bend test (ISO 1519), reported as the minimum cracking-free mandrel diameter (mm). Abrasion resistance of coatings was measured by falling sand test (ASTM D968, results in L mil^−1^) on three coated specimens per formulation. FE-SEM imaging was performed using a JEOL JSM-IT800 at an accelerating voltage of 3 kV with a secondary electron detector (SED). Powder samples were mounted on conductive carbon tape. Both the powder samples and the fracture surfaces of the nanocomposite materials were sputter-coated with platinum to reduce charging during observation. The working distances are indicated in the corresponding micrographs. EDX analysis was performed in area scan mode. Spectra were acquired at an accelerating voltage of 15 kV, a working distance of 10 mm, and a probe current of 1 nA, with a live time of 60 s per spectrum. Evaluate the thermal properties of materials on a thermogravimetric analyzer TGA 209F (Netzsch, Germany) with a heating rate of 10 °C min^−1^, under a nitrogen purge gas flow of 20 mL min^−1^, using Al_2_O_3_ crucibles, from room temperature to 700 °C. Dynamic mechanical analysis (DMA-1, Mettler Toledo) was performed using rectangular bar specimens (30 × 5 × 1.5 mm) at 1 Hz and heating rate of 4 °C min^−1^ from 30 to 170 °C and a strain of 0.1% in a tension mode, recording storage modulus (*E′*) and tan *δ*. Coating thickness was measured at ≥5 locations per coupon using a magnetic induction gauge (ISO 2178). Surface gloss was measured at 60° geometry (ISO 2813) with ≥5 readings per sample.

## 3 Result and discussion

### 3.1. Characterization of TiO_2_@SiO_2_ hybrid nanoparticles

The successful synthesis of the TiO_2_@SiO_2_ (TS) hybrid nanoparticles *via* the Stöber sol–gel method was confirmed by FE-SEM morphological analysis, FT-IR spectroscopy, and EDX elemental analysis (area scan mode). Fig. 1 shows the FE-SEM micrographs of neat TiO_2_ and TS nanoparticles.

**Fig. 1:**
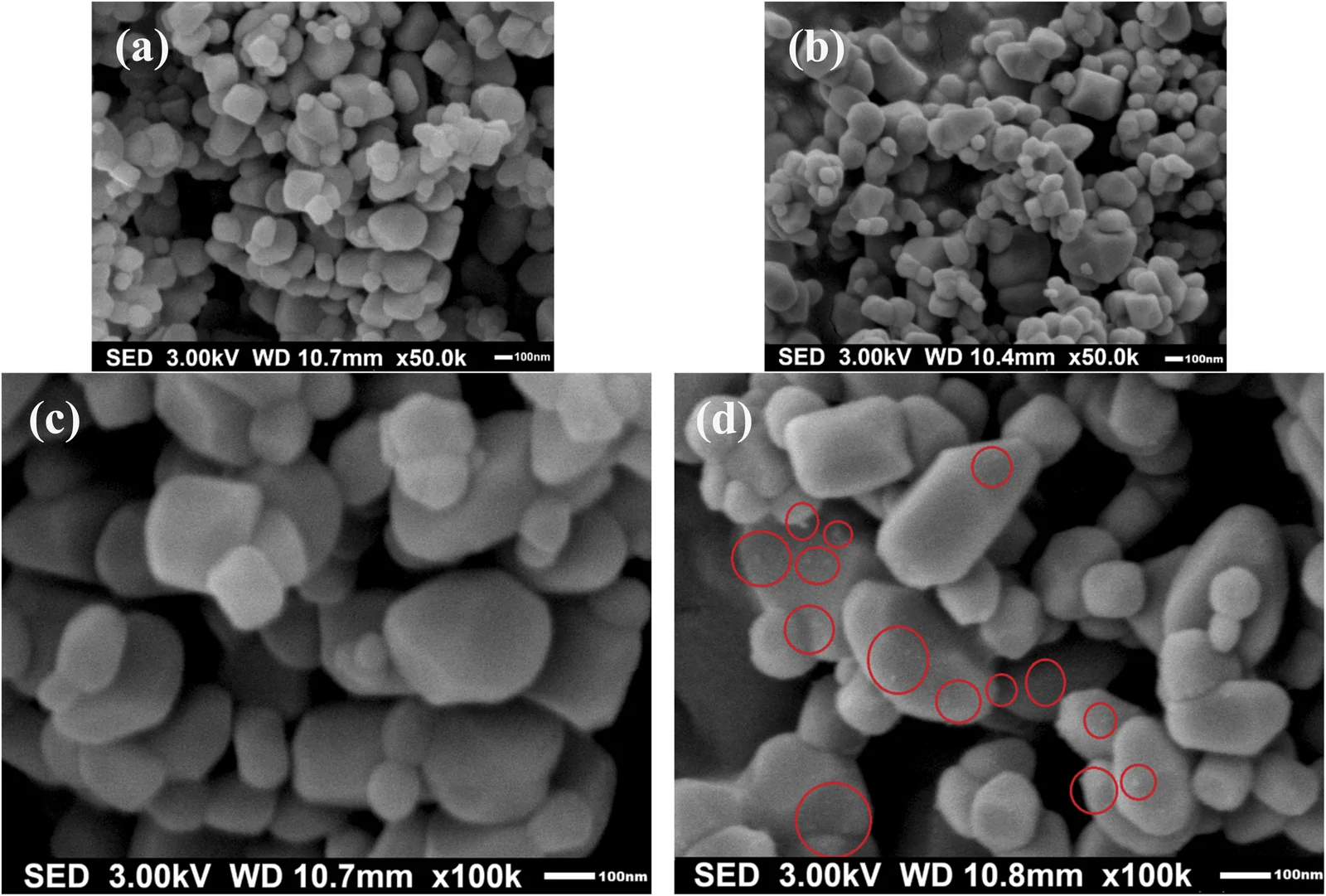
FE-SEM micrographs of (a and c) neat TiO_2_ nanoparticles and (b and d) TiO_2_@SiO_2_ (TS) hybrid nanoparticles synthesized *via* the Stöber method.

The neat TiO_2_ nanoparticles (Fig. 1a) exhibit irregular, angular shapes with pronounced inter-particle agglomeration forming clusters of 150–300 nm, driven by the strong van der Waals attractive forces characteristic of high-surface-energy oxide nanoparticles.^17^ At the magnifications employed (Fig. 1b), the TS nanoparticles present an overall particle size and agglomeration tendency broadly comparable to those of neat TiO_2_. This similarity is expected because the SiO_2_ shell was deposited without additional organic surface modification: the abundant free silanol (–Si–OH) groups on the SiO_2_ surface are themselves highly polar and readily form strong inter-particle hydrogen bonds between adjacent TS nanoparticles, sustaining agglomeration comparable in extent to that observed for neat TiO_2_.^12^ The TS nanoparticles appear markedly darker than neat TiO_2_ under identical beam and detector conditions. One possible contribution to this difference is atomic-number-dependent electron backscattering, since SiO_2_ shell (Si, *Z* = 14) generally has a lower backscattered electron yield than TiO_2_ core (Ti, *Z* = 22) under comparable conditions. Backscattered electrons can also generate secondary electrons and thereby contribute to the signal recorded by a secondary electron detector.^25^ The SiO_2_ shell may therefore contribute to the observed contrast difference. However, the current images were acquired from Pt-coated samples, and the contrast depends on factors such as surface topography, working distance, and signal acquisition conditions. It is impossible to isolate the contribution of the SiO_2_ shell from these factors. Therefore, this explanation is only hypothetical, and the observed brightness difference alone—while potentially a contributing factor—is insufficient to confirm the presence of the SiO_2_ shell.

Furthermore, at very high magnification (×100 k; Fig. 1d), a distinctive flake-like or scale-like surface micro-texture is clearly resolved on the TS particle surface, entirely absent on the smooth surface of the neat TiO_2_ particles (Fig. 1c). This scaly texture is assigned to the deposition of the amorphous SiO_2_ layer onto the TiO_2_ core during the Stöber sol–gel condensation reaction, providing direct morphological evidence for the successful coating of the TiO_2_ surface with SiO_2_.

Fig. 2 shows the FT-IR spectra of neat TiO_2_ and TS nanoparticles with diagnostic absorption bands labeled I–VII. In the spectrum of neat TiO_2_, a broad, intense absorption band spanning approximately 500–900 cm^−1^ is assigned to Ti–O stretching and Ti–O–Ti lattice vibrations of the rutile phase.^26^ Two bands at approximately 3300 cm^−1^ and 1630 cm^−1^ are attributed to O–H stretching of surface hydroxyl groups and physisorbed water, and to H–O–H bending of adsorbed water, respectively.^22,26^ The absence of any Si–O–Si features in the 800–1200 cm^−1^ region confirms that the neat TiO_2_ precursor is free of siliceous species.

**Fig. 2:**
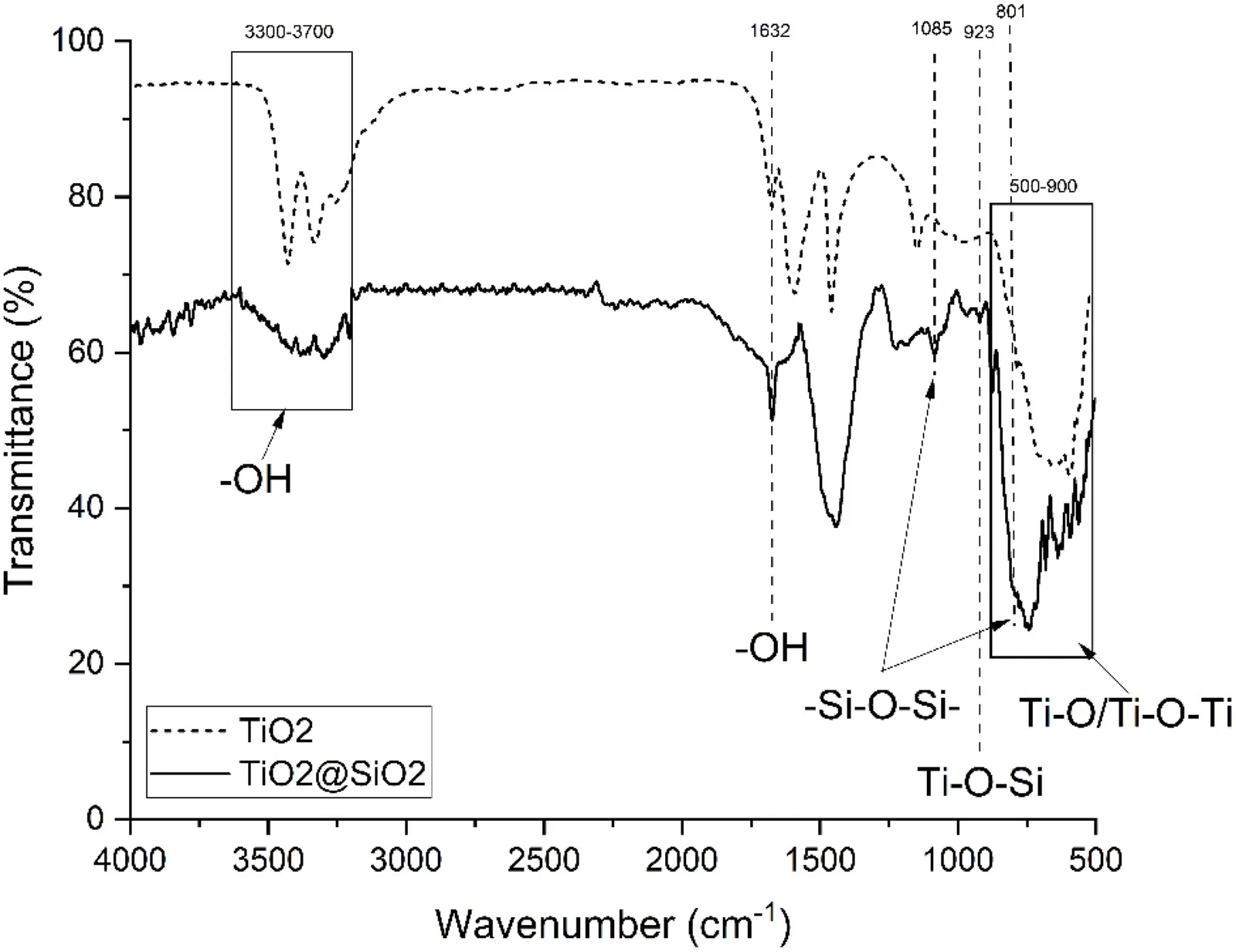
FT-IR spectra of neat TiO_2_ nanoparticles and TiO_2_@SiO_2_ hybrid nanoparticles.

The FT-IR spectrum of the TS nanoparticles retains all characteristic bands of neat TiO_2_ while displaying new features that confirm SiO_2_ shell deposition. The dominant new feature is a strong broad band at approximately 1080–1090 cm^−1^, characteristic of the asymmetric Si–O–Si stretching vibration in amorphous silica.^22^ Band at approximately 800–810 cm^−1^ is assigned to the symmetric Si–O–Si stretching.^22^ Of particular diagnostic significance is a peak at 923 cm^−1^, attributed to stretching of interfacial Ti–O–Si heterolinkages formed by condensation of surface Ti–OH (TiO_2_ core) and Si–OH groups during TEOS hydrolysis;^22^ this result proves that the SiO_2_ shell is covalently bonded to the TiO_2_ core. The simultaneous appearance of two new peaks at 1085 and 923 cm^−1^ in the TS spectrum provide clear FT-IR evidence for the formation of a covalently bonded TiO_2_@SiO_2_ core–shell structure.

The elemental composition of the TS nanoparticles was further confirmed by EDX spectroscopy (Fig. 3). The spectrum displays peaks attributable exclusively to Ti, O, and Si, confirming the absence of significant inorganic impurities.^18,25^ The Ti Kα and Ti Kβ peaks at ∼4.51 keV and ∼4.93 keV, respectively, confirm the TiO_2_ core.^18^ The O Kα signal at ∼0.52 keV corresponds to oxygen in both TiO_2_ and SiO_2_. The Si Kα peak at ∼1.74 keV (ref. 18 and 25) provides direct elemental evidence for silicon incorporation. The detection of Si by EDX, together with the Si–O–Si and interfacial Ti–O–Si vibrations identified by FT-IR, provides complementary evidence for the successful formation of TiO_2_@SiO_2_ hybrid nanoparticles.

**Fig. 3:**
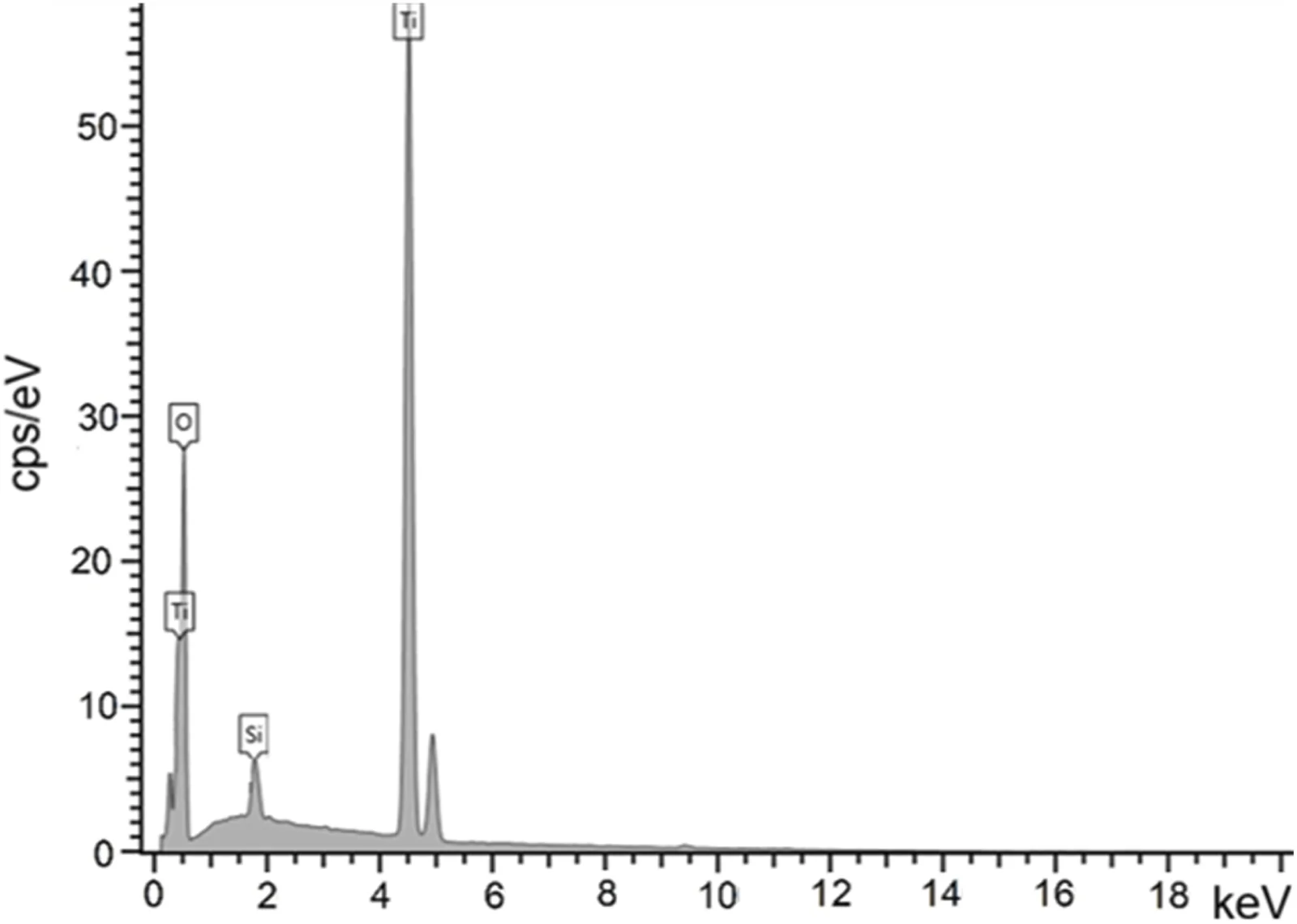
EDX spectrum of TiO_2_@SiO_2_ (TS) nanoparticles confirming the presence of Ti, O, and Si elements.

### 3.2. Mechanical properties and abrasion resistance of PA12/PPO nanocomposite coatings

The tensile strength, Young's modulus, flexibility (mandrel bend test), and falling sand abrasion resistance of the PA12/PPO blend and its nanocomposite coatings are compiled in Table 1. Samples are named as T-1, T-3, T-5 (1, 3, 5 wt% neat TiO_2_) and TS-1, TS-3, TS-5 (1, 3, 5 wt% TiO_2_@SiO_2_ hybrid nanoparticles). Flexibility was evaluated by a mandrel bend test, in which the FBC-coated steel coupon is bent over cylindrical mandrels of decreasing diameter; the result is reported as the diameter (mm) of the smallest mandrel over which the coating survives without cracking. A lower mandrel diameter indicates a more flexible, ductile coating. Tensile specimens were prepared by hot-pressing the melt-compounded powder at 250 °C to replicate the thermal history of the FBC sintering cycle, and abrasion resistance was evaluated on FBC-coated steel coupons by the falling sand method (ASTM D968), expressed in liters of sand per mil (L mil^−1^) of coating thickness.

**Table 1:** Mechanical properties of PA12/PPO and its nanocomposite coatings

Sample	Tensile strength (MPa)	Young's modulus (GPa)	Mandrel bend flexibility (mm)	Abrasion resistance (L mil^−1^)
PA12/PPO	29.1 ± 0.5	1.35 ± 0.02	<2	560.7 ± 16.8
T-1	30.4 ± 0.8	1.42 ± 0.04	<2	608.6 ± 21.3
T-3	31.6 ± 0.7	1.53 ± 0.05	2	690.6 ± 20.7
T-5	29.7 ± 2.1	1.61 ± 0.09	4	649.6 ± 54.2
TS-1	31.2 ± 0.7	1.46 ± 0.04	<2	635.9 ± 22.1
TS-3	34.1 ± 0.6	1.64 ± 0.04	2	793.2 ± 19.8
TS-5	31.9 ± 1.5	1.71 ± 0.07	2	731.6 ± 43.9

As shown in Table 1, both nanoparticle series produce a monotonic increase in Young's modulus with increasing filler content, while tensile strength and abrasion resistance follow a bell-shaped dependence with an optimum at 3 wt%, and coating flexibility (mandrel bend result) progressively decreases with increasing filler content. Regarding tensile strength and stiffness, at 1–3 wt% loading the well-dispersed nanoparticles create an extensive polymer–filler interfacial area that promotes stress transfer from the matrix to the rigid inorganic phase and restricts segmental chain mobility, raising both tensile strength and modulus.^27^ At 5 wt%, the mean tensile strength decreases from 31.6 to 29.7 MPa in the TiO_2_ series and from 34.1 to 31.9 MPa in the TS series compared with the corresponding 3 wt% formulations. One possible explanation is increased nanoparticle agglomeration at the higher loading, which could reduce stress-transfer efficiency and introduce local stress concentrations that promote premature failure. Meanwhile, the continued increase in Young's modulus is consistent with the increasing content of rigid inorganic filler.^28^ The larger standard deviations in tensile strength, Young's modulus, and abrasion resistance observed for T-5 and TS-5 may reflect greater microstructural heterogeneity at the higher filler loading, potentially associated with nanoparticle agglomeration. This interpretation is consistent with literature suggesting that agglomeration effects may increase the variability of mechanical test data.^27,29,30^ Across all loadings, the TS series achieves superior tensile strength and abrasion resistance than the TiO_2_ series at equivalent loading. The TS-3 sample achieves the highest tensile strength of 34.1 MPa and the highest abrasion resistance of 793.2 L mil^−1^, compared with 31.6 MPa and 690.6 L mil^−1^ for T-3. This systematic superiority may partly arise from hydrogen bonding interactions between the silanol groups (Si–OH) of the SiO_2_ shell and the amide groups (–NH–CO–) of the PA12 backbone, consistent with the well-established interfacial bonding mechanism reported for silica–polyamide nanocomposite systems,^31–33^ potentially enabling more efficient stress transfer and more uniform coating microstructure, as discussed in relation to the FE-SEM observations in Section 3.3.^20^ Hydrogen bonding is proposed here as a literature-supported interfacial mechanism rather than an interaction directly verified in the present nanocomposites.

The mandrel bend test results reveal a progressive decrease in coating flexibility with increasing filler content, reflecting the stiffening effect of the inorganic nanoparticles on the PA12/PPO matrix. The neat PA12/PPO coating and both 1 wt% nanocomposites (T-1, TS-1) pass the <2 mm mandrel without cracking, demonstrating good ductility at low filler loadings. At 3 wt%, both T-3 and TS-3 pass the 2 mm mandrel, indicating a modest but acceptable reduction in flexibility consistent with the higher Young's moduli of 1.53 and 1.64 GPa, respectively. A critical divergence emerges at 5 wt%: T-5 requires a 4 mm mandrel diameter, indicating a marked loss of flexibility, whereas TS-5 retains acceptable flexibility at the 2 mm mandrel. This distinction may reflects differences in filler dispersion and interfacial adhesion at high loading: the agglomerated TiO_2_ clusters in T-5 could create rigid, poorly bonded micro-inclusions that promote cracking under bending and initiate interfacial delamination under the mandrel bend loading, substantially compromising coating ductility. In TS-5, more effective filler–matrix interactions may help distribute the bending load more evenly, allowing the coating to retain flexibility even if agglomeration occurs at this loading level. For FBC coating applications on components with curved geometries—such as pipe fittings, automotive fuel line connectors, and dishwasher baskets—preservation of coating flexibility at the 2 mm mandrel level is an important practical criterion, making TS-5 preferable to T-5 at high filler loadings.

The monotonically increasing Young's modulus across both series—from 1.35 GPa for neat PA12/PPO to 1.71 GPa for TS-5— may have implications for the upstream FBC powder preparation process. In FBC technology, the polymer blend is typically reduced to powder by cryogenic ball milling or cryogenic impact grinding at temperatures below the glass transition temperature of the material, where it becomes brittle and fractures into particles of the target size range (80–200 µm).^1,11^ The higher Young's modulus at 5 wt% TS (1.71 GPa, increased 26.7% *vs.* PA12/PPO) may be relevant to powder production in light of two literature-informed considerations: (i) the material fractures may be more likely to fracture readily under impact, potentially facilitating particle size reduction;^24^ however (ii) cryogenic milling can also produce irregular, angular particles with a broad size distribution,^24^ potentially degrading the flowability of the resulting FBC powder.^34^ These potential processing considerations warrant further investigation alongside the measured advantages of the TS-3 formulation—which achieves the best combination of tensile strength, abrasion resistance, and coating flexibility while maintaining a moderate modulus of 1.64 GPa. The proposed connection between the measured stiffness trends and milling behavior is a literature-based hypothesis that has not been experimentally evaluated in this study.

### 3.3. Morphology of PA12/PPO polymer nanocomposite coatings

The FE-SEM micrographs of the fracture surfaces of the T-3 and TS-3 samples – selected as representative formulations at 3 wt% filler loading based on their superior mechanical properties compared to all formulations tested in Section 3.2 – at magnifications of ×5000 (Fig. 4a and b) and ×30 000 (Fig. 4c and d) provide a qualitative basis for discussing the fracture morphology and possible interfacial interactions in relation to the mechanical properties presented in Section 3.2.

**Fig. 4:**
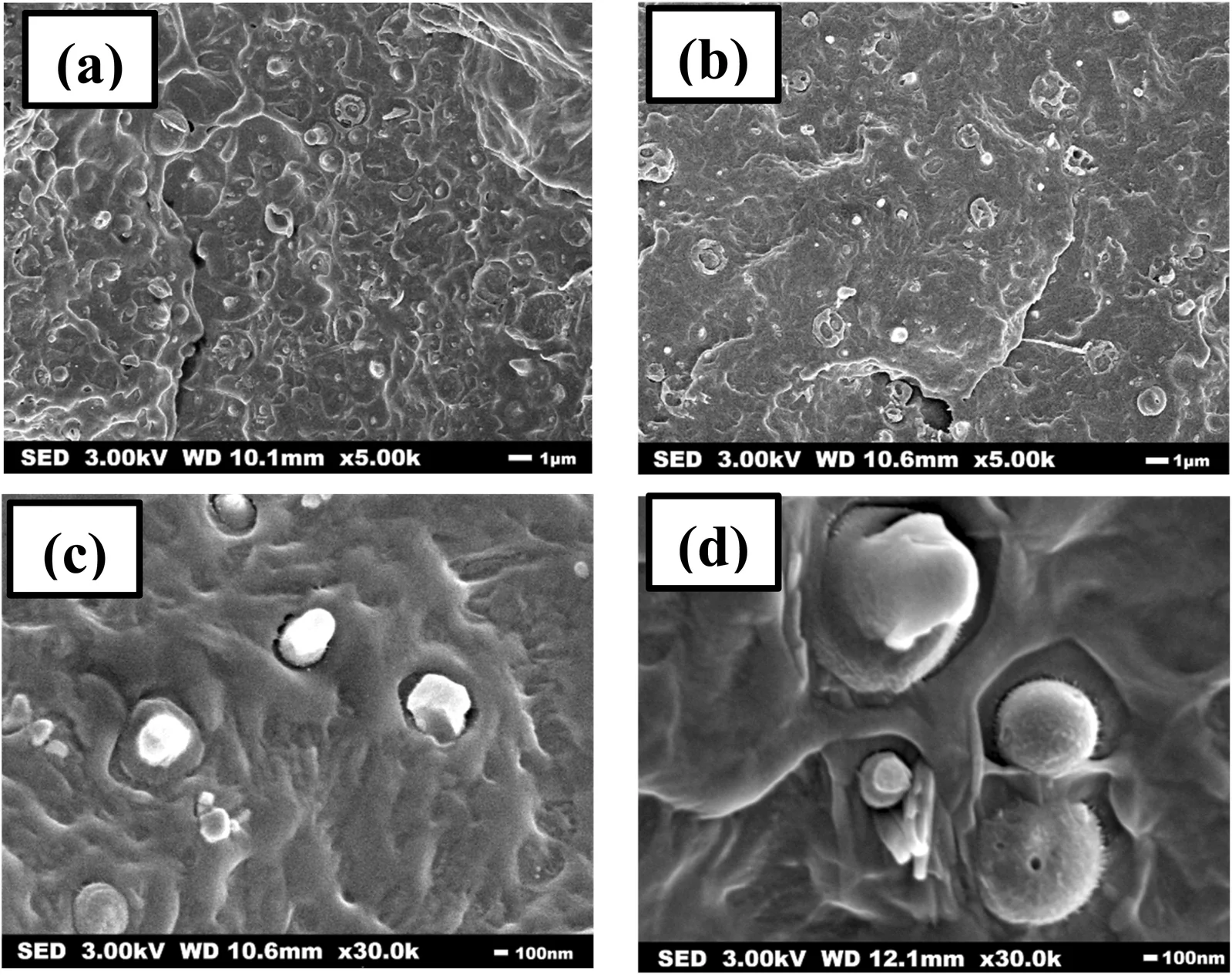
FE-SEM micrographs of fracture surfaces of (a and c) PA12/PPO/3%TiO_2_ and (b and d) PA12/PPO/3%TiO_2_@SiO_2_ FBC coatings at ×5k and ×30k magnification, respectively.

At the overview magnification of ×5,000, both T-3 (Fig. 4a) and TS-3 (Fig. 4b) exhibit the rough, undulating fracture surfaces characteristic of a two-phase, partially miscible PA12/PPO blend. In both micrographs, discrete spherical or near-spherical features that appear as protruding globules or cavity-like regions are distributed across the fracture surface; these are assigned to PPO-rich dispersed domains on morphological grounds, whose separation from the PA12 matrix during fracture leaves either protruding globules or hemispherical pull-out cavities depending on the local fracture path and interfacial interactions. Irregular fracture ridges and small particulate features were excluded from this morphological assignment. In the T-3 fracture surface (Fig. 4), the PPO domains appear as isolated, smooth-walled spheres with clear circumferential gaps at the PA12/PPO phase boundary, consistent with local separation at the polymer–polymer interface during fracture. In the TS-3 fracture surface (Fig. 4b), the boundaries between some PPO domains and the surrounding matrix appear less sharply demarcated, which may reflect local differences in interfacial fracture behavior.

The ×30k micrographs provide additional qualitative information for the different interfacial behavior of TiO_2_ and TS nanoparticles within the PA12/PPO matrix. In Fig. 4c (T-3, ×30k), the TiO_2_ nanoparticles were observed to be nearly spherical objects with bright, smooth surfaces. Their contrast with the surrounding polymer may partly reflect atomic-number-dependent electron backscattering from Ti-containing regions, including its contribution to secondary-electron generation.^25^ However, this compositional contribution cannot be separated from the effects of surface topography, Pt coating, and signal-collection conditions, as discussed in Section 3.1. Critically, each TiO_2_ particle in Fig. 4c is surrounded by a distinct circumferential void or debonding gap between the particle surface and the polymer matrix. This interfacial separation is consistent with limited local adhesion between the neat TiO_2_ surface and the PA12/PPO matrix: under tensile or impact loading, stress concentration at such interfacial defects triggers early debonding and void coalescence, limiting the mechanical reinforcing efficiency of TiO_2_ nanoparticles and potentially contributing to the comparatively modest tensile strength improvement in the T-3 sample.^28^

The ×30 000 micrograph of the TS-3 sample (Fig. 4d) presents a distinctly different morphology. Some rounded features in Fig. 4d appear darker than the bright features observed in Fig. 4c. The SiO_2_ shell may contribute to this difference by altering the contribution of backscattering – a phenomenon dependent on atomic number – to the detected signal. More importantly, the surface of the TS particles in Fig. 4d exhibits a distinctively rough, fibrous, or filament-like texture at the particle-matrix contact zone, in marked contrast to the smooth, featureless TiO_2_ particle surfaces in Fig. 4c. This fibrous interfacial texture may be interpreted as polymer chain fibrils anchored to and stretched from the SiO_2_ shell surface during fracture: the surface silanol (–Si–OH) groups of the SiO_2_ shell may form interfacial hydrogen bonds with the amide groups of the PA12 backbone, potentially tethering polymer chains to the particle surface. When the composite is fractured, these tethered chains could elongate and draw into fibrils before ultimately rupturing, creating the thread-like morphology observed and potentially contributing to energy dissipation in the process.^20^ Furthermore, no circumferential debonding gap is observed around the TS particles in Fig. 4d – the polymer matrix wraps continuously and conformally around each particle – supporting the interpretation that the SiO_2_ shell promotes stronger filler-matrix adhesion and more effective stress transfer that efficiently transfers mechanical load between the polymer chains and the inorganic core.

Selective localization of TS nanoparticles at the PA12/PPO interface is considered here as a possible contribution to morphological stabilization, depending on the relative wettability of the nanoparticle surface by the two polymer phases.^9,20^ If TS nanoparticles localize at the PA12/PPO interface, they could function as solid-particle emulsifiers in a Pickering-type stabilization mechanism:^35^ the nanoparticles are partially wetted by each polymer phase and create a steric barrier that impedes the coalescence of PPO droplets during melt compounding, potentially refining and stabilizing the PPO dispersed-phase morphology.^9^ The SiO_2_ shell is particularly well-suited to this interfacial role because its surface silanol groups may provide strong hydrogen-bonding interactions with the amide groups of PA12 on one side of the interface, while the less polar aromatic surface of the PPO phase on the other side interacts more weakly—creating an asymmetric wettability that drives nanoparticle migration and anchoring to the interfacial zone.

Collectively, the FE-SEM morphological analysis suggests that the SiO_2_ shell of the TS hybrid nanoparticles may influence nanoparticle localization, enhances surface interaction with the PA12/PPO matrix better than the neat TiO_2_ nanoparticles, potentially contributing to superior mechanical reinforcement at the same filler content.

### 3.4. Thermogravimetric analysis of materials

The TGA and DTG curves of the neat PA12/PPO blend material and its T-3 and TS-3 nanocomposites are shown in Fig. 5, and characteristic thermal decomposition parameters are compiled in Table 2.

**Fig. 5:**
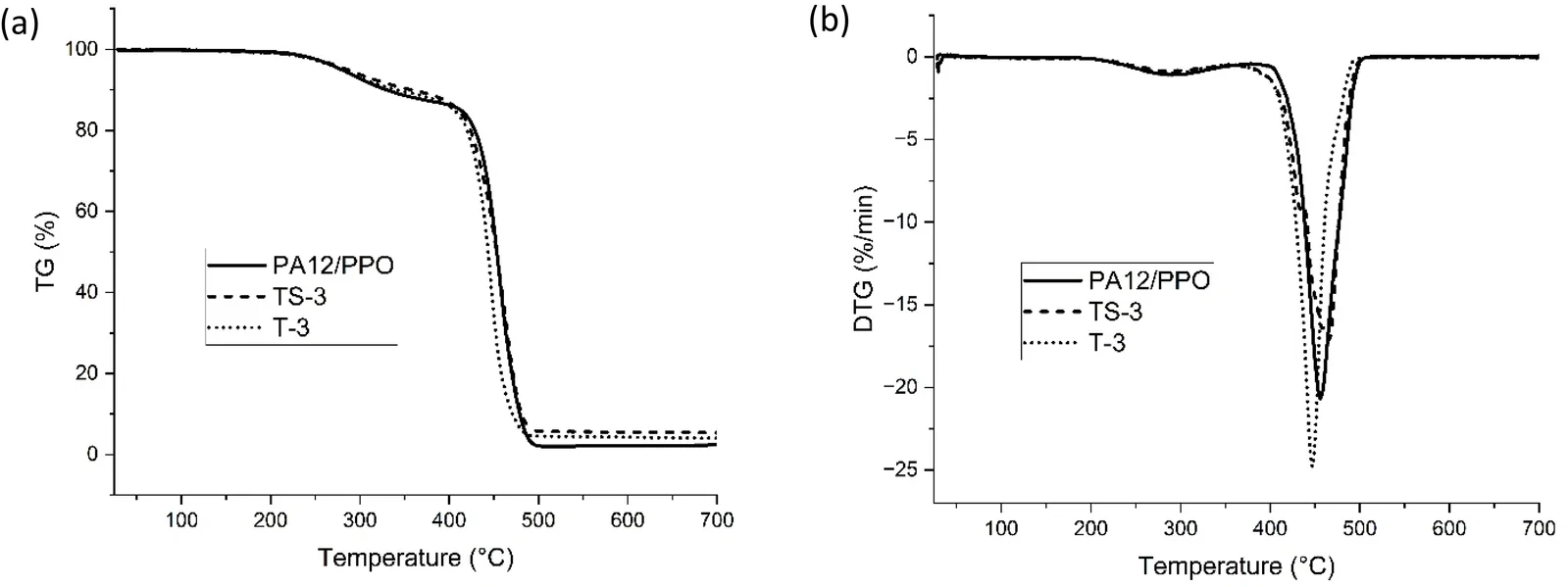
Characteristic parameters for the thermal decomposition process of material samples: (a) TGA curves; (b) DTG curves.

**Table 2:** Thermogravimetric parameters of PA12/PPO and its 3% nanocomposite coating materials (N_2_ atmosphere, 10 °C min^−1^)

Sample	*T*_10%_ (^o^C)	*T*_max_ (^o^C)	Residual char rate (%)
PA12/PPO	332.6	456.4	0.29
T-3	340.9	446.9	3.98
TS-3	358.3	465.2	5.34

All three samples undergo a single-stage thermal decomposition event in N_2_. The T-3 sample displays a notable and scientifically significant bifurcated behavior: its *T*_10%_ (340.9 °C) is marginally higher than that of the neat PA12/PPO blend (332.6 °C), indicating a slight delay in the onset of mass loss attributable to the physical barrier effect of TiO_2_ nanoparticles impeding the outward diffusion of early pyrolysis volatiles. However, the peak decomposition temperature (*T*_max_) of T-3 is 446.9 °C, which is 9.5 °C lower than that of the neat PA12/PPO blend (456.4 °C). This decrease in *T*_max_ indicates that, once thermal decomposition is initiated, it proceeds more rapidly – and reaches its maximum rate at a lower temperature – in the presence of neat TiO_2_ nanoparticles than in the unfilled blend. This behavior may be attributed to the Lewis acid surface activity of TiO_2_: the exposed Ti^4+^ cation sites on the TiO_2_ surface may act as Lewis acid catalysts that interact with the electron-rich amide groups (–CO–NH–) of the PA12 backbone, facilitating C–N and C–C bond homolysis and lowering the activation energy for chain scission under thermal stress.^36,37^ The net result is a dual effect: TiO_2_ slightly delays the onset of degradation but subsequently accelerates the decomposition process once chain scission is initiated, with the consequence that the deposited FBC coating undergoes more rapid thermal breakdown at elevated temperatures than either the unfilled blend or the TS-3 nanocomposite. This thermal catalytic effect of TiO_2_ on polymer matrices has been independently documented for polyamide and other engineering thermoplastic systems.^5,38^

In contrast, the TS-3 nanocomposite exhibits a decisive improvement in both onset and peak thermal stability. Its *T*_10_% of 358.3 °C represents a 25.7 °C improvement over the neat PA12/PPO blend and a 17.4 °C improvement over T-3, while its *T*_max_ of 465.2 °C is 8.8 °C higher than that of the neat blend and 18.3 °C higher than T-3. The superior performance of TS-3 relative to T-3 in both parameters is consistent with three possible protective contributions of the SiO_2_ shell in the TS hybrid nanoparticles: (i) it may physically block the Lewis acid Ti^4+^ surface sites of the TiO_2_ core, potentially reducing their contribution to the chain-scission process proposed to account for the T_max decrease in T-3; (ii) it may provide a more effective and thermally stable inorganic barrier network around the TiO_2_ core, potentially impeding volatile diffusion,^18,39^ and (iii) it may promote more homogeneous nanoparticle dispersion and improved interfacial interaction with the PA12/PPO matrix, potentially favoring a more effective barrier to volatile transport compared with the TiO_2_-filled system.

The residual mass at 700 °C in N_2_ increases monotonically from 0.29 wt% (neat PA12/PPO) to 3.98 wt% (T-3) and 5.34 wt% (TS-3). The higher residue for TS-3 compared with T-3 reflects the additional contribution of the thermally stable, non-combustible SiO_2_ shell: whereas neat TiO_2_ remains as the only inorganic residue in T-3, the SiO_2_ shell in TS-3 also forms a thermally stable char-like silica network upon polymer burnout. At temperatures above 700 °C, the SiO_2_ shell undergoes partial sintering and densification, creating a rigid, low-permeability inorganic network that acts as an additional passive barrier against the thermal decomposition of any residual polymer species underneath.^39^ From a practical FBC coating perspective, the higher residue in TS-3 also implies a larger non-combustible mass fraction that will retain structural integrity at extreme temperatures, potentially improving the fire-resistance performance of the coating on metal substrates.

### 3.5. Dynamic mechanical analysis of materials

Fig. 6a shows the temperature dependence of storage modulus (*E′*) and Fig. 6b the tan *δ* curves of the neat PA12/PPO coating material and its T-3 and TS-3 nanocomposites, with key DMA parameters listed in Table 3. DMA probes the viscoelastic response of the material under dynamic oscillatory loading, providing direct information on the rigidity of the polymer network and the mobility of polymer chains in each phase.

**Fig. 6:**
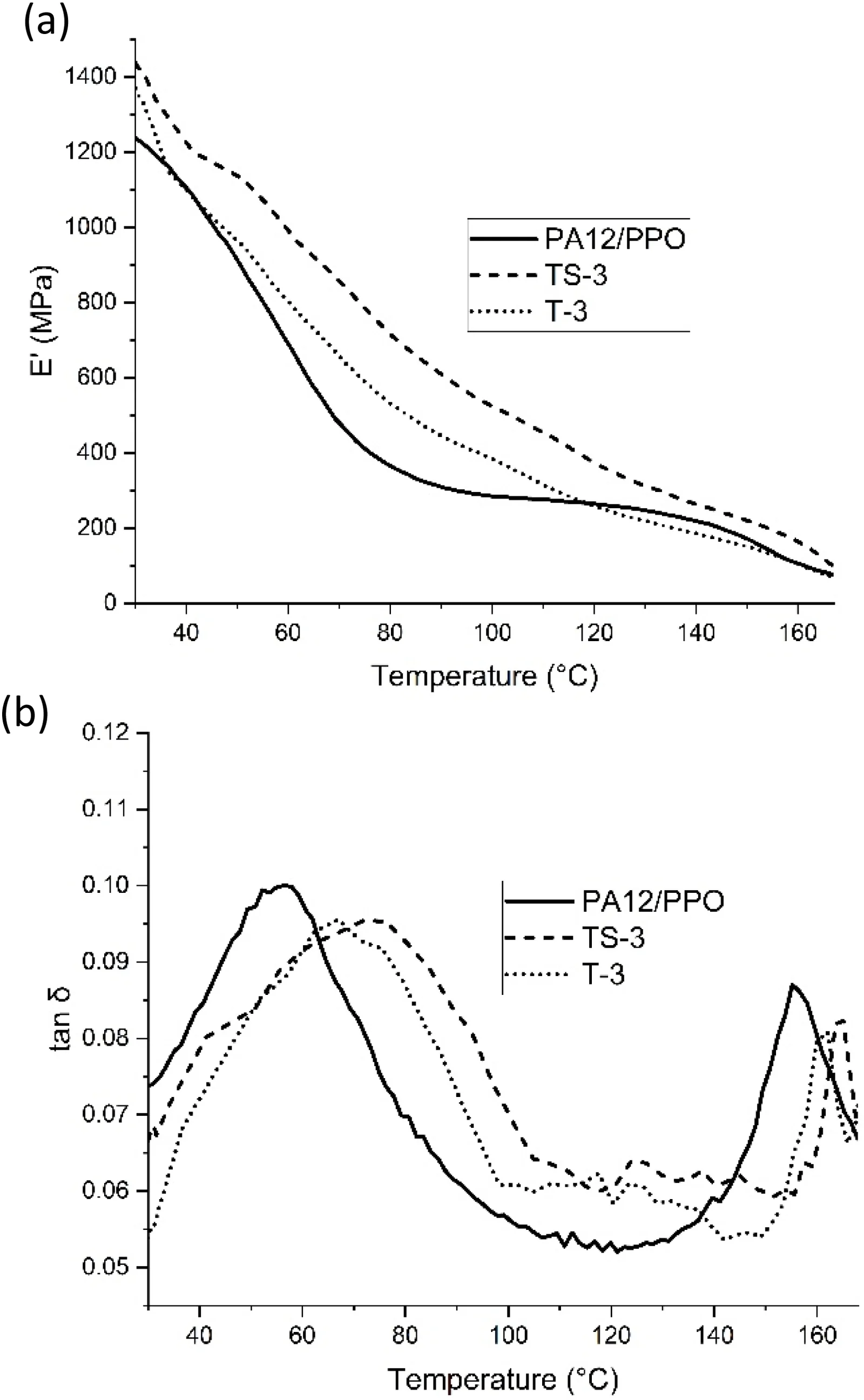
Storage modulus (a) and tan *δ* (b) as a function of temperature for neat PA12/PPO coating material and its nanocomposites with 3 wt% TiO_2_ (T-3) and 3 wt% TiO_2_@SiO_2_ (TS-3).

**Table 3:** DMA parameters of PA12/PPO and its 3% nanocomposite coating materials

Sample	T_g_1__ (PA12 phase, °C)	T_g_2__ (PPO phase, °C)	*E*′ at 30 °C (MPa)
PA12/PPO	56.2	155.6	1241
T-3	67.0	161.8	1370
TS-3	71.9	165.1	1438

The storage modulus (*E′*) at 30 °C increases progressively from 1241 MPa for the neat PA12/PPO blend to 1370 MPa for T-3 and 1438 MPa for TS-3. This monotonic stiffening trend is quantitatively consistent with the Young's modulus data measured by quasi-static tensile testing in Section 3.2, confirming that both test methodologies consistently capture the reinforcing effect of the nanoparticles on the PA12/PPO matrix. The higher *E′* of TS-3 relative to T-3 at equivalent loading is consistent with stronger polymer–filler interfacial interactions potentially involving the silanol groups of the SiO_2_ shell, which restrict local segmental chain mobility more effectively than the weakly adhered neat TiO_2_ particles.^32,33^ The increased dynamic stiffness of the TS-3 nanocomposite coating may be relevant to coatings on load-transmitting contact surfaces, where limiting small elastic deformations is desirable.^40^

Regarding the connection to FBC powder processability, the progressively higher *E′* of the nanocomposite blends implies a harder, stiffer material at the cryogenic milling temperature used for FBC powder preparation. A harder milled material fractures more cleanly under impact, potentially yielding narrower particle size distributions and higher milling throughput at equivalent specific energy input—a process advantage that is directly relevant to the FBC powder manufacturing step. Moreover, the increased stiffness of the cured coating (reflected in *E′*) means that the deposited FBC films are better able to withstand scratching and deformation during handling of coated components prior to end use.^3^

The tan *δ* curves (Fig. 6b) reveal two well-resolved relaxation peaks for all three samples, corresponding to the glass transition of the PA12-rich phase (T_g_1__) and the PPO-rich dispersed phase (T_g_2__). The presence of two distinct, well-separated relaxation events in both nanocomposites confirms that the partially miscible two-phase character of the PA12/PPO blend is preserved upon nanoparticle incorporation at 3 wt%, without macrophase separation or complete miscibilization.^10^

For the T-3 nanocomposite, both *T*_g_ values shift positively relative to the neat blend: ΔT_g_1__ = +10.8 °C (67.0 *vs.* 56.2 °C) and ΔT_g_2__ = +6.2 °C (161.8 *vs.* 155.6 °C). For the TS-3 nanocomposite, the shifts are even larger: ΔT_g_1__ = +15.7 °C (71.9 *vs.* 56.2 °C) and ΔT_g_2__ = +9.5 °C (165.1 *vs.* 155.6 °C). These consistently larger shifts in the TS-3 system confirm that TS nanoparticles more effectively restrict segmental mobility in both polymer phases potentially through the stronger interfacial hydrogen bonds involving by the SiO_2_ shell silanol groups, as discussed in Sections 3.2 and 3.3. The T_g_1__ shift for TS-3 is particularly significant in the FBC context: a higher glass transition temperature of the PA12 phase in the deposited coating means the coating surface retains its solid-state rigidity to higher temperatures, delaying the onset of surface softening and deformation under thermal loading in service. Equally important, a higher *T*_g_ of the nanocomposite powder blend means that the powder particles are less prone to inter-particle adhesion and block formation during storage or transport at ambient temperatures, improving the shelf life and consistency of the FBC powder feedstock.^3^

### 3.6. Coating thickness and surface gloss

Coating thickness and surface gloss are key technological performance parameters for FBC-deposited coatings, governing protective function and aesthetic appearance, respectively. In FBC, thickness uniformity is controlled by the powder's melt viscosity, flowability, and particle size distribution;^1^ a lower standard deviation in thickness indicates more homogeneous powder flow and melt coalescence across the substrate.^41^ Surface gloss at 60° is controlled by microscale surface roughness: a smoother coating surface produces higher gloss values.^41^Table 4 compiles the coating thickness and surface gloss for the PA12/PPO blend, T-3, and TS-3 coatings.

**Table 4:** Coating thickness and surface gloss (60°) of PA12/PPO, T-3, and TS-3 FBC coatings on mild steel substrates

Sample	Coating thickness (µm)	Gloss
PA12/PPO	305 ± 34	67.7
T-3	291 ± 46	57.1
TS-3	318 ± 21	70.5

As shown in Table 4, the T-3 coating exhibits a higher standard deviation in thickness than the neat PA12/PPO coating, reflecting greater uniformity disruption by the less homogeneous dispersion of nano TiO_2_ compared to TS hybrid nanoparticles that are proposed to locally alter melt viscosity during sintering, potentially creating discontinuities in film spreading—a mechanism consistent with the established understanding that nanoparticle aggregation can introduce local viscosity heterogeneity in polymer melt systems.^15,16,27^ In contrast, the TS-3 coating achieves the lowest thickness variability of the three systems – the proposed contributions of nanoparticle dispersion and interfacial interactions discussed in Section 3.3 – because uniformly distributed TS particles produce a spatially consistent melt viscosity that enables even spreading and coalescence of the polymer film.^1^

Regarding surface gloss, the T-3 coating shows a lower 60° gloss value than both the neat PA12/PPO blend and TS-3. This reduction arises from two mechanisms: agglomerate clusters not fully encapsulated by the polymer melt create surface asperities that scatter incident light;^14^ and bare TiO_2_ particles with a very high refractive index (*n* ≈ 2.5) act as strong light-scattering centers near the coating surface, contributing to a matte appearance.^17,41^ The TS-3 coating achieves the highest gloss of the three systems. Well-dispersed TS nanoparticles fully encapsulated in the polymer melt do not form protruding surface asperities; instead they fill sub-micron surface valleys and smooth the coating topography. Moreover, the SiO_2_ shell reduces the refractive index contrast between nanoparticle and polymer matrix compared with bare TiO_2_, decreasing per-particle light-scattering efficiency. These improvements in thickness uniformity and surface gloss—achieved without compromising the mechanical and thermal properties established in Sections 3.2–3.5—confirm the practical advantage of the TS-3 formulation for FBC applications where both protective performance and coating appearance are specified.^1,14,41^

## 4 Conclusions

In summary, the incorporation of TiO_2_@SiO_2_ hybrid nanoparticles into a PA12/PPO (90/10 wt%) blend by melt compounding was found to improve tensile strength, abrasion resistance, and thermal stability relative to the unfilled blend; furthermore, the formulation containing 3 wt% of hybrid nanoparticles (TS-3) provides greater mechanical reinforcement compared to the formulation using original TiO_2_ nanoparticles at the same loading (T-3). The mean tensile strength of the TS-3 sample reached 34.1 MPa, compared with 29.1 MPa for the unfilled blend and 31.6 MPa for T-3 sample. Furthermore, the temperature at 10% mass loss for this sample showed an improvement over the neat PA12/PPO blend and was 17.4 °C higher than that of the T-3 sample, while retaining coating flexibility at the 2 mm mandrel level. The SiO_2_ shell may contribute to these improvements through several complementary effects: limiting direct contact between the TiO_2_ surface and the polymer matrix, potentially reducing surface-mediated degradation; providing silanol groups capable of hydrogen bonding with PA12; and influencing nanoparticle localization and polymer–filler adhesion. The fibrous fracture texture observed in TS-3 is consistent with differences in interfacial adhesion relative to T-3, while possible localization at the PA12/PPO phase boundary could contribute to interfacial stabilization. The higher thermal stability and residual mass of TS-3 are also compatible with possible barrier and residue-retention contributions from the silica-containing filler. These mechanisms provide a tentative interpretation of the measured improvements, including the lower coating thickness variability and higher surface gloss of TS-3.

Beyond the measured coating properties themselves, the higher glass transition temperatures and stiffness of the TS-containing blends may have implications for FBC powder preparation and handling. Changes in low-temperature fracture behavior could affect cryogenic milling, particle size distribution, and particle shape, with subsequent consequences for powder flow and fluidization. These potential processing effects remain literature-informed considerations requiring experimental evaluation. The present results nevertheless identify PA12/PPO/TS as a promising system for further development of FBC coatings and motivate closer examination of the relationships between interfacial modification, powder preparation, and coating performance.

## Author contributions

Conceptualization, Nguyen Vu Giang, Pham The Long; data analysis, Pham The Long, Tran Huu Trung, Mai Duc Huynh, Nguyen Huu Dat, Nguyen Tien Minh; methodology, Pham The Long, Nguyen Vu Giang, Nguyen Huu Dat; project administration, Nguyen Thi Thu Trang; validation, Luong Nhu Hai, Nguyen Vu Giang, Nguyen Thi Ngoc Quynh, and Pham The Long; writing – original draft, Nguyen Vu Giang, Pham The Long; writing – review & editing, Nguyen Thi Thu Trang, Pham The Long, Hoang Thi Van An and Luong Nhu Hai. All authors have read and agreed to the published version of the manuscript.

## Conflicts of interest

The authors declare that they have no conflicts of interest.

## Data Availability

All data generated or analyzed during the current study are included in the article. The datasets used and/or analyzed in the current study are available from the corresponding author upon reasonable request. There are no restrictions apply to the data, and all data can be made available to the scientific community for future research upon request.
